# Comparative Analysis of Endoscopic and Microscopic Myringoplasty

**DOI:** 10.1055/s-0045-1809929

**Published:** 2025-10-07

**Authors:** Gabriel Souza Mares, Raquel Pinto Coelho Souza Dias, Felipe Costa Neiva, Cidia Vasconcellos

**Affiliations:** 1 Otology Division, Otorhinolaryngology Service, Hospital do Servidor Público Estadual “Francisco Morato de Oliveira” (HSPE-FMO), Instituto de Assistência Médica ao Servidor Público Estadual (IAMSPE), São Paulo, SP, Brazil

**Keywords:** myringoplasty, endoscopy, microscopy, otology

## Abstract

**Introduction:**

Myringoplasty is a surgical procedure aimed at reconstructing the tympanic membrane, restoring acoustic protection to the round window, and improving sound conduction to enhance hearing, and prevent recurrent infections or otorrhea. This procedure can be performed either through microscopy or endoscopy.

**Objective:**

To compare the outcomes of endoscopic myringoplasty and microscopic myringoplasty.

**Methods:**

Data were retrospectively collected from the electronic medical records of patients who underwent myringoplasty either through microscopy or videoendoscopy at Hospital do Servidor Público Estadual de São Paulo (HSPE) between December 2015 and December 2020. We evaluated the therapeutic success rate, defined as tympanic membrane closure, the variation in the pre- and postoperative speech recognition threshold (SRT), and the incidence of immediate and late complications in each group.

**Results:**

We included 118 patients: 64 underwent microscopic myringoplasty (group 1) and 54, videoendoscopic myringoplasty (group 2). The groups were comparable in terms of mean age, sex, and body mass index (BMI). The surgical success rates were similar between the 2 groups: group 1: 70.3%; and group 2: 75.9% ( *p* = 0.494). Group 1 presented a significant improvement in the mean postoperative hearing thresholds (30.0 ± 14.9) compared to the mean preoperative levels (41.0 ± 16.3) ( *p* < 0.001), while group 2 did not present a statistically significant improvement (from 37.8 ± 14.7 preoperatively to 32.0 ± 20.7 postoperatively; *p* = 0.284).

**Conclusion:**

Microscopic and endoscopic myringoplasty yielded similar tympanic membrane closure rates. However, the videoendoscopic procedures resulted in lower SRT reduction, while the microscopic procedures resulted in a higher rate of immediate postoperative complications.

**Level Of Evidence:**

3

## Introduction

Persistent tympanic perforations, lasting longer than 3 months, are one of the main features of chronic otitis media (COM), with irreversible changes to the middle-ear mucosa. 1 Such changes can lead to recurrent middle-ear infections, the development of cholesteatomas, and hearing loss, 2 3 4 5 6 generating great expense for the health care system. 7

Myringoplasty is a surgical procedure that aims at reconstructing the tympanic membrane (TM), restoring acoustic protection to the round window, and restoring the mechanisms that conduct sound, improving hearing and preventing recurrent infections/otorrhea. 8 9 It can be performed through microscopy or endoscopy.

Myringoplasty by microscopy (MM) was one of the first procedures described to address tympanic perforations, and it can be performed via the retroauricular, transcanal, or endaural routes. 10 The retroauricular incision enables greater visibility of the surgical field and is therefore the globally preferred approach. The transcanal route is usually restricted to those patients with small tympanic perforations and a wide external acoustic meatus (EAM). 10 11 Although MM results in high rates of therapeutic success, it often requires scalp shaving and a retroauricular incision, in the case of the retroauricular approach. 12

Myringoplasty by endoscopy (ME), on the other hand, does not require extensive incisions; therefore, it may be less invasive than MM. The main difference between the two techniques is the visibility of the surgical field. While in MM there is a limitation to vision due to the narrower segment of the EAM, in ME this segment is overcome, enabling a wider view. 13 14 On the other hand, in MM, binocular vision provides a better sense of depth, and the surgeon can use both hands to hold the surgical instruments, which does not occur in ME, in which it is necessary to hold the optician with one hand. 14

The rates of TM closure by endoscopic myringoplasty range from 80 to 100%, and by microscopic myringoplasty, from 83 to 100%. 10 11 12 However, although both techniques are widespread, to date, few studies 15 16 comparing their clinical outcomes and postoperative complications have been conducted. Thus, the purpose of the present paper is to evaluate the efficacy of TM closure applying these different methods, using the experience of a tertiary care hospital in the city of São Paulo, Brazil, as an overview.

## Methods

The current work, as part of the otology research line of the Otolaryngology Department of Hospital do Servidor Público Estadual de São Paulo (HSPE), was approved by the Ethics and Medical Research Committee of the Instituto de Assistência Médica ao Servidor Público Estadual de São Paulo (IAMSPE) on August 16, 2021, under opinion number 4.909.550 and CAE (Board of Economic Matters) 5007373721.7.0000.5463.

The present was a retrospective, longitudinal, observational study following a historical cohort model of analysis of medical records of patients undergoing unilateral myringoplasty by endoscopy and by microscopy in a tertiary care hospital in São Paulo (HSPE) between December 2015 and December 2020.

The patients included in the study were those who underwent unilateral myringoplasty, in the period, due to chronic non-suppurative and non-cholesteatomatous otitis media that lead to complications (presence of persistent tympanic perforation without persistent suppuration, that is, lasting longer than 3 months), with regular postoperative follow-up of at least 6 months at the Otorhinolaryngology Department of HSPE. All surgeries were performed by third-year residents, oriented by four experienced otologists of the institution.

The data were collected directly and exclusively from the electronic medical records via the MV soul electronic system (MV Informática Nordeste Ltda), based on the surgical records of the Otorhinolaryngology Department of HSPE, and stored in digital tables.

The following variables were analyzed: age, sex and comorbidities of the patients, and surgical technique applied. Then, we analyzed the outcome of the following variables throughout 6 months of outpatient visits: surgical success/residual TM perforation (characterized by the persistence of the perforation within 6 months of the postoperative follow-up); changes in speech recognition threshold (SRT, evaluated between the preoperative audiometry and the last postoperative audiometry); and late or immediate postoperative complications (considered as complications that occurred within the first 24 hours after the procedure).

For comparative purposes, the patients were divided into two groups according to the surgical method applied (videoendoscopy or microscopy). Patients with a history of otologic surgery in the ear studied; those with other otologic pathologies, such as cholesteatoma or persistent suppuration (lasting longer than 3 months), which could interfere in the postoperative result; and subjects with insufficient data in their medical records or who did not undergo adequate follow-up in the 6 months following surgery were excluded from the study.

The variables evaluated were presented in tables through absolute and relative frequency distribution. The normality of the variables was tested by the Shapiro-Wilk test, and the associations were tested by the Pearson's Chi-squared test or Fisher's exact test, when necessary.

The statistical significance of the differences in means among the quantitative variables were verified using the paired and unpaired Student's *t* -test. All analyses were performed at a 5% significance level; therefore, the results were considered statistically significant when the *p* value was lower than 0.05, always considering two-tailed alternative hypotheses. The information collected formed a database developed in an Excel for Windows (Microsoft Corp.) spreadsheet, and the statistical analysis was performed using the STATA 11 SE software (StataCorp LLC).

## Results

From December 2015 to December 2020, a total of 176 myringoplasties were performed at Hospital do Servidor Público Estadual de São Paulo (HSPE).

After applying the inclusion and exclusion criteria, we obtained a sample of 118 patients, who were divided into 2 groups according to the method used in the surgery: 64 patients (54.2%) via microscopy ( **group 1** ) and 54 patients (45.8%) by videoendoscopy ( **group 2** ).

Most patients were female, constituting 62.5% of group 1 (40 patients) and 57.4% of group 2 (31 patients), with no statistically relevant difference ( *p* = 0.573). Neither did the mean age of the two groups show statistical significance ( *p* = 0.710): 46.9 years in group 1 (range: 6–77 years) and 45.7 years in group 2 (range: 9–71 years). In both groups, most patients were overweight, with a mean body mass index (BMI) of 28.7 kg/m ^2^ and 28.4 kg/m ^2^ in groups 1 and 2 respectively ( *p* = 0.887) ( Table 1 ).

**Table 1:** Demographic characteristics and body mass index of groups 1 and 2

Variables	Group 1	Group 2	*p*
Mean age (years)	46.9 ± 17.3	45.7 ± 17.7	0.710 ^a^
Gender: n (%)			
* Male*	40 (60.5)	31 (57.4)	0.573 ^b^
* Female*	24 (47.5)	23 (42.6)	
Mean body mass index (kgm ^2^ )	28.7 ± 5.2	28.4 ± 9.6	0.887 ^a^

**Notes:** Group 1: patients who underwent microscopic myringoplasty; group 2: patients submitted to videoendoscopy myringoplasty. ^a^ Independent samples *t* -test. ^b^ Fisher's exact test.

Most of the patients analyzed (69 patients; 58.5%), did not present comorbidities, while 49 patients (41.5%) presented at least 1 comorbidity. In both groups, the most prevalent comorbidity was systemic arterial hypertension (SAH), with 18 patients (29.5%) in group 1 and 17 patients (32.7%) in group 2, followed by dyslipidemia (DLP), with 10 patients in group 1 (16.4%) and 5 patients (9.6%) in group 2, and diabetes mellitus (DM), with 5 patients (8.2%) in group 1 and 8 patients (15.4%) in group 2, all without statistical significance ( *p* > 0.05). The prevalence of smoking was 19.7% in group 1 (12 patients) and of 14.9% in group 2 (7 patients), without statistically significant differences ( *p* = 0.518) ( Table 2 ).

**Table 2:** Prevalence of comorbidities and smoking in groups 1 and 2

Variables	Group 1	Group 2	*p*
Comorbidity: n (%)			
* Systemic arterial hypertension*	18 (29.5)	17 (32.7)	0.718 ^a^
* Dyslipidemia*	10 (16.4)	5 (9.6)	0.290 ^a^
* Diabetes mellitus*	5 (8.2)	8 (15.4)	0.233 ^a^
Smoking: n (%)	12 (19.7)	7 (14.9)	0.518 ^a^

**Notes:** Group 1: patients who underwent microscopic myringoplasty; group 2: patients submitted to videoendoscopy myringoplasty. ^a^ Fisher's exact test.

**Table 3:** Perforation size in patients in groups 1 and 2

Variables	Group 1	Group 2	*p*
Mean perforation size (%) ± SD	48.0 ± 25.5	35.8 ± 24.6	0.055 ^a^

**Notes:** Group 1: patients who underwent microscopic myringoplasty; group 2: patients submitted to videoendoscopy myringoplasty. ^a^ Independent samples *t* -test.

The choice of surgical technique used in each case varied according to the surgeon's experience/preference. There was no statistically significant difference in the size of the perforation between the two groups ( Table 3 ). The surgical team is composed of four otologic assistant surgeons and residents in training. In group 2, the access was 100% transcanal, with variations in the type of graft used: 12 patients with temporal fascia graft (22.2%), 41 patients with cartilage graft (75.9%), and 1 patient with fat graft (1.9%). In group 1, in 19 patients the access was transcanal (29.7%), 13 with cartilage graft (20.3%), 4 with fat graft (6.3%), and 2 with temporal fascia graft (3.1%). In the other 45 patients in group 1, the access was retroauricular, with temporal fascia graft (70.3%) ( Table 4 ).

**Table 4:** Access routes and type of graft used in groups 1 and 2

Variables	Group 1	Group 2
Access route: n (%)		
* Transcanal myringoplasty*	15 (29.7)	54 (100.0)
* Retroaural myringoplasty*	45 (70.3)	0 (0.0)
Type of graft: n (%)		
* Temporal fascia*	47 (73.4)	12 (22.2)
* Cartilage*	13 (20.3)	41 (75.9)
* Fat*	4 (6.3)	1 (1.9)

**Notes:** Group 1: patients who underwent microscopic myringoplasty; group 2: patients submitted to videoendoscopy myringoplasty.

**Table 5:** Values of *p* for statistical causality of the variables correlated to the presence of residual perforation (therapeutic failure)

	Residual perforation		*p*
Variable	Yes	No	
	n = 32	n = 86	
Mean age (Years)	44.8 ± 18.6	46.9 ± 17.0	0.612 ^a^
Mean body mass index (kgm ^2^ )	28.9 ± 10.5	28.3 ± 5.1	0.711 ^a^
Mean preoperative speech recognition threshold (dB)	40.4 ± 13.0	39.2 ± 16.6	0.803 ^a^
Mean postoperative speech recognition threshold (dB)	40.6 ± 18.9	27.5 ± 16.2	0.005 ^a^
Sex: n (%)			
* Female*	19 (59.4)	52 (60.5)	0.914 ^b^
* Male*	13 (40.6)	34 (39.5)	
Type of graft: n (%)			
* Temporal fascia*	18 (56.3)	41 (47.7)	0.882 ^b^
* Cartilage*	13 (40.6)	41 (47.7)	
* Fat*	1 (3.1)	4 (4.6)	
Immediate postoperative complication: n (%)			
* No*	23 (71.9)	71 (82.6)	0.200 ^b^
* Yes*	9 (28.1)	15 (17.4)	
Late postoperative complication: n (%)			
* No*	18 (56.3)	70 (81.4)	0.005 ^b^
* Yes*	14 (43.7)	16 (18.6)	
Smoking: n (%)			
* No*	23 (74.2)	66 (85.7)	0.155 ^b^
* Yes*	8 (25.8)	11 (14.3)	
Diabetes mellitus: n (%)			
* No*	29 (90.6)	76 (88.4)	0.728 ^b^
* Yes*	3 (9.4)	10 (11.6)	
Systemic arterial hypertension: n (%)			
* No*	24 (75.0)	59 (68.6)	0.759 ^b^
* Yes*	8 (25.0)	27 (31.4)	
Dyslipidemia: n (%)			
* No*	30 (93.7)	73 (84.9)	0.199 ^b^
* Yes*	2 (6.3)	5 (15.1)	

**Notes:** Group 1: patients who underwent microscopic myringoplasty; group 2: patients submitted to videoendoscopy myringoplasty. ^a^ Independent samples *t* -test. ^b^ Fisher's exact test.

**Table 6:** Incidence of immediate and late postoperative complications in groups 1 and 2

Postoperative complications	Group 1	Group 2	*p*
Immediate: n (%)	21 (32.8)	3 (5.5)	< 0.001 ^a^
Late: n (%)	18 (28.1)	12 (22.2)	0.463 ^a^

**Notes:** Group 1: patients who underwent microscopic myringoplasty; group 2: patients submitted to videoendoscopy myringoplasty. ^a^ Fisher's exact test.

**Fig. 1:**
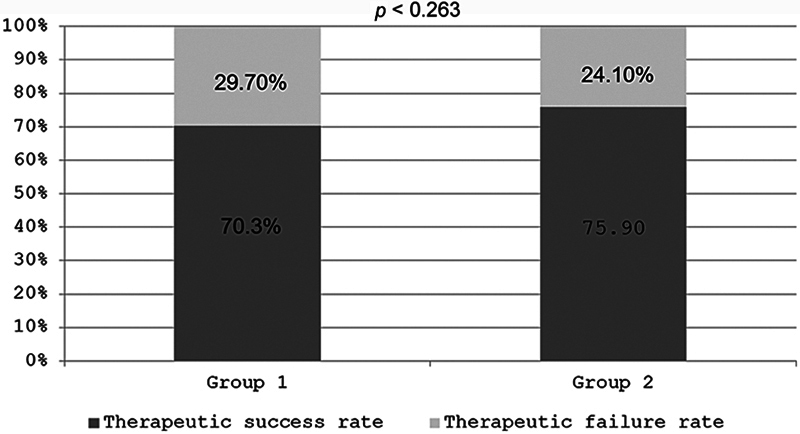
Therapeutic success and failure rates for tympanic membrane (TM) closure in groups 1 and 2.

The main criterion for therapeutic failure in myringoplasty is the persistence of tympanic perforation in the first 6 months after surgery. 9 20 The rate of TM closure rate was 70.3% in group 1 and of 75.9% in group 2, with no statistically significant differences ( *p* = 0.263) ( Figure 1 ). Comparing patients with therapeutic failure and success, we observed that, in those with maintenance of the tympanic perforation, the postoperative db was higher (40.6 dB versus 27.5 dB respectively), which was statistically significant ( *p* = 0.005). In these same patients, a higher prevalence of late complications was also observed (27.3% versus 19.8% respectively; *p* = 0.007). No causality relationship was observed regarding the tympanic perforation rate and other variables such as comorbidity, graft type, access route, age, gender, nor smoking ( Table 5 ).

**Fig. 2:**
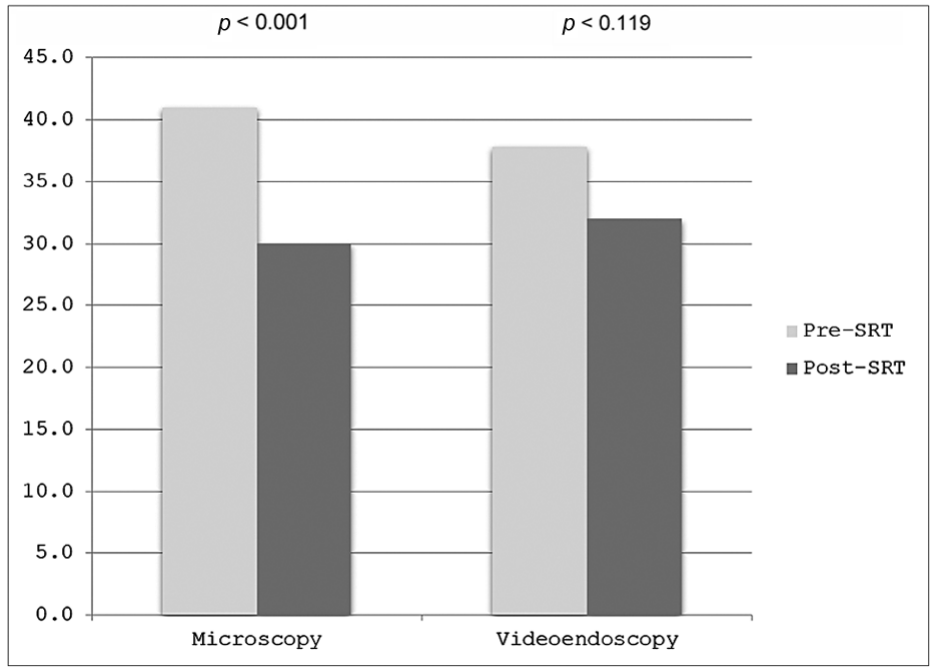
Graph with pre- and postoperative speech recognition thresholds (SRTs) of patients who underwent myringoplasty by microscopy and videoendoscopy.

In the current study, we also compared the variation in SRT between the pre- and postoperative audiometries in the two groups. In group 1, the mean preoperative SRT was of 41.0 ± 16.3 dB, with a mean reduction to 30.0 ± 14.9 dB postoperatively, with a statistically significant difference ( *p* < 0.001). In group 2, we also observed a reduction in the mean SRT, from 37.8 ± 14.7 dB preoperatively to 32.0 ± 20.7dB post-operatively, but without a statistically significant difference ( *p* = 0.119) ( Figure 2 ). Neither were there statistically significant differences between the mean preoperative SRTs of the 2 groups ( *p* = 0.284), nor regarding the comparidon of the postoperative SRTs of the 2 groups ( *p* = 0.649).

Regarding surgical complications, 21 patients in group 1 (32.1%) presented them in the immediate postoperative period (up to 24 hours after the procedure), while only 3 patients (5.6%) in group 2 presented such complications, with a statistically significant difference ( *p* < 0.001) *(* Table 6 ). The most common immediate complications were otorrhea (9 cases), followed by dizziness (7 cases), otalgia (4 cases), dysgeusia (3 cases), and tinnitus (1 case) ( Table 7 ).

**Table 7:** Types of immediate and late postoperative complications by group

Postoperative complications	Group 1	Group 2
Immediate: n (%)		
* Dysgeusia*	2 (9.5)	1 (33.3)
* Otalgia*	4 (19.1)	0 (0.0)
* Otorrhea*	7 (33.3)	2 (66.7)
* Dizziness*	7 (33.3)	0 (0.0)
* Tinnitus*	1 (4.8)	0 (0.0)
Late: n (%)		
* Permanent dysgeusia*	1 (5.6)	0 (0.0)
* Incision granuloma*	1 (5.6)	0 (0.0)
* Acute otitis externa*	8 (44.4)	7 (53.8)
* Acute otitis media*	2 (11.1)	3 (25)
* Otalgia*	1 (5.6)	1 (7.7)
* Otomycosis*	1 (5.6)	0 (0.0)
* Dry mouth syndrome*	0 (0.0)	1 (7.7)
* Dizziness*	4 (22.2)	1 (7.7)

**Notes:** Group 1: patients who underwent microscopic myringoplasty; group 2: patients submitted to videoendoscopy myringoplasty.

Evaluating late the postoperative complications, we observed an incidence of 28.1% (18 patients) in group 1 and of 22.2% (12 patients) in group 2, with no statistically significant difference ( *p* = 0.463) ( Table 6 ). The most frequent late complications were acute otitis externa (AEO; (15 cases), followed by acute otitis media (5 cases), and persistent dizziness (5 cases) ( Table 7 ). No patients required postoperative emergency readmission, and no deaths were observed during the study period. Analyzing the complications per type of surgical access, a higher incidence of immediate postoperative complications was found in patients submitted to the retroauricular surgical access (33.3%) in relation to transcanal access (12.3%), with a statistical difference ( *p* = 0.006). The same pattern was not observed for late complications ( *p* = 0.265) ( Table 8 ).

**Table 8:** Incidence of immediate and late postoperative complications according to the surgical access used in myringoplasty

Postoperative complications	Type of surgical access		*p*
	Transcanal	Retroaural	
Immediate: n (%)	9 (12.3)	15 (33.3)	0.006 ^a^
Late: n (%)	16 (21.9)	14 (31.1)	0.265 ^a^

**Note:**^a^ Fisher's exact test.

## Discussion

Microscopic myringoplasty is considered the standard surgery for the closure of tympanic perforations in patients with chronic non-suppurative and non-cholesteatomatous otitis media, and it has been widely spread since its popularization in the 1950s by Wullstein and Zollner. 4 17 Otologic endoscopic surgery has gained space since 1990, 18 aiming at avoiding unnecessary incisions and dissections of soft tissue, and at providing better visualization of sites with difficult access in the middle ear. 17 19 In the current study, from the 118 patients selected, 64 (54.2%) underwent MM while 54 patients (45.8%) underwent ME, showing a similar frequency in the use of both methods in HSPE.

The presence of residual tympanic perforation after myringoplasty is a criterion for therapeutic failure. 9 20 The rates of TM closure rates observed in groups 1 and 2 of the current study showed no statistically significant differences, which is consistent with other studies and systematic reviews comparing MM and ME. 8 9 21 22 The absolute values of TM closure rates at HSPE (70.3% in group 1 and 75.1% in group 2), however, were somewhat lower than those observed in large centers, 21 22 23 where rates ranging from 80 to 100% are observed. This increased rate of therapeutic failure, or presence of residual perforation, may be associated with the teaching profile of the institution where the study was conducted, in which the lack of experience of surgeons in training (residents) may be considered, by some authors, 24 a risk factor for surgical failure. Compared to the rates observed in other teaching hospitals, 25 26 however, the rates found in the current study were similar.

The type of graft used (temporal fascia graft, fat graft, cartilage graft, among others) and the choice of surgical approach (transcanal or retroauricular) in myringoplasties are topics for extensive discussions in literature. Regarding TM closure, in the present study, there was no association between the type of graft nor access route used and the surgical outcome observed. This finding is consistent with the current literature, 27 28 29 30 in which both accesses and grafts present very similar efficacy, and their choice depends on the surgeon's experience and best indication for the case. Some studies, 30 31 32 33 however, differ from such data and show higher rates of TM closure when tragal cartilage is used compared to temporal fascia.

Some studies 20 34 35 36 37 point to the presence of smoking, multiple comorbidities, and Eustachian tube dysfunction as risk factors for therapeutic failure in myringoplasty. Therapeutic failure, that is, failure of the graft incorporation to the TM, would be related to a change in the microvascularization and pressure regulation of the tympanic cavity, reducing graft fixation. 34 In the current study, the two groups had a similar profile, with no statistically significant difference regarding age, gender, BMI, smoking, or presence of comorbidities. In none of the groups was a causal relationship observed regarding any comorbidity, smoking, age or sex and the primary outcome, which disagrees with some recent studies, 34 36 which point to smoking as a major risk factor for an unfavorable outcome.

Hearing rehabilitation and improvement in the postoperative audiometric parameters in patients undergoing myringoplasty are outcomes well established in the literature. 11 13 In the present study, in both groups, a reduction in the postoperative SRT was observed compared to the preoperative SRT, but only in group 1 was this reduction statistically significant. Such a finding may be related to the larger portion of patients in whom cartilage graft was used in group 2 (75.9%; 41 patients), leading to a lower SRT reduction due to a greater mass effect of cartilage compared to temporal fascia, the predominant graft in group 1 (73.4%; 47 patients). 29 33 This finding, however, is not a consensus in the literature, in which some studies 27 28 35 have observed no statistically significant difference in pre- and postoperative audiometry according to the type of graft used.

The main postoperative complications of tympanoplasty are the presence of otalgia, otorrhea, transient dysgeusia, and dizziness. 38 39 40 Other rarer complications are peripheral facial palsy and sensorineural hearing loss. 40 They are associated with direct manipulation of middle-ear and EAM structures, and their incidence may be related to the status and size of the perforation of the operated ear, the functionality of the Eustachian tube, the patient's comorbidities, the operative time, and the surgeon's experience. 38 40 In the current study, immediate complications, considered as those occurring within 24 hours of the surgery, were more prevalent in group 1 compared to group 2, with a statistically significant difference ( *p* < 0.001). There was no statistically significant difference between the two groups regarding late complications. These findings may be related to the more invasive nature of the retroauricular access, which constituted the main access in MM (70.3%; 45 patients), and the increased incidence of complications occurs due to the longer operative time and the need for more extensive incisions and plane dissections. 9 16 40

The main limitation to the current study is its retrospective design, as the surgical technique was determined by the surgeon, introducing a potential selection bias. While a randomized clinical trial would better mitigate this bias, its implementation in tympanoplasty remains challenging 9 16 . Additionally, despite the efforts to control for confounders, TM perforation size was not explicitly accounted for. Given its examiner-dependent and inherently subjective nature, this could introduce variability. 6 21 However, the absence of a statistically significant difference in perforation size between the two study groups minimizes its potential impact on the results ( Table 3 ).

## Conclusion

The present study showed no difference in the efficacy of MM or ME, with similar TM closure rates in both groups. The main difference observed between the two methods was a lower SRT reduction in patients submitted to ME and a higher incidence of immediate postoperative complications in patients submitted to MM, but these findings may be related to the type of graft and access used respectively.
